# Early Detection of Multiple Sclerosis Using Spectroscopic Epstein–Barr Virus Biosensors Based on Infection Mechanism Mimicry

**DOI:** 10.1021/acs.analchem.5c03964

**Published:** 2025-09-15

**Authors:** Ülkü Anık, Nil Su Çaylayık

**Affiliations:** † Sensors, Biosensors and Nano-Diagnostic Systems Laboratory, Research Laboratory Center, 52986 Mugla Sitki Kocman University, Mugla 48000, Turkey; ‡ Faculty of Science, Chemistry Department, 229199 Mugla Sitki Kocman University, Mugla 48000, Turkey

## Abstract

The association between Epstein–Barr virus (EBV) and multiple sclerosis has made the asymptomatic detection of EBV very significant. In this study, a spectroscopic/colorimetric EBV biosensor was developed for practical and early screening by replicating the virus’s natural infection mechanism. In order to accomplish this, gold nanoparticles (AuNPs) were functionalized with the B cell surface protein CD21, thereby mimicking the native host cell receptor. The interaction between CD21@AuNP and the EBV surface glycoprotein gp350 was then obtained spectroscopically through absorbance measurements, which was accompanied by a visible color change. Experimental parameters, including the incubation temperature and time, were systematically optimized to enhance the assay performance. Under optimized conditions, the biosensor exhibited a linear detection range of 10–100 ng/mL, with a limit of detection of 0.0279 ng/mL, a limit of quantification of 0.0848 ng/mL, and a relative standard deviation of 0.848%. Validation with 1:100 diluted saliva samples obtained from healthy volunteers further confirmed the sensor’s high sensitivity and robustness, demonstrating its strong potential for translation into real-world diagnostic applications.

## Graphical abstract

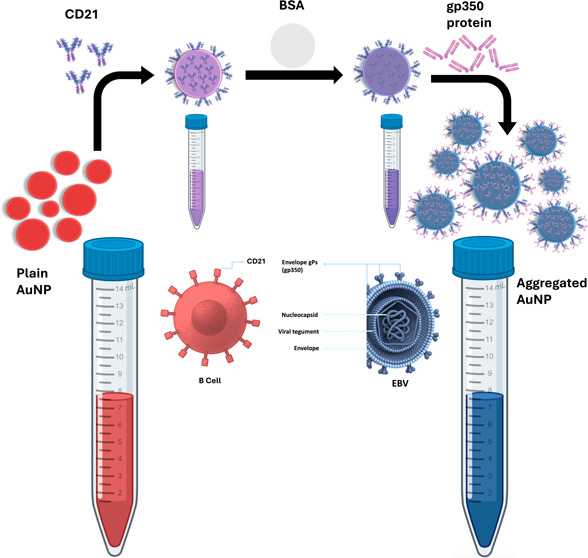

## Introduction

Epstein–Barr virus (EBV), a type of human γ herpesvirus 4, is highly infectious and remains in the host’s body for life. Although it is often considered harmless, EBV has been linked to several types of cancers and autoimmune diseases, including multiple sclerosis (MS).^1^ Studies have shown that primary EBV infection—commonly known as infectious mononucleosis (IM)—and high titers of EBV antibodies are associated with the development of MS, even when IM occurred many years before the appearance of the first MS symptoms.^2,3^

Meanwhile, MS is a heterogeneous autoimmune disease in which the body’s immune system—particularly autoreactive lymphocytes—mistakenly attacks the myelin sheath that protects nerve fibers in the central nervous system. This immune response leads to the destruction of myelin in multiple areas, leaving behind scar tissue, known as sclerosis. As a result of this damage, the transmission of electrical signals to and from the brain is disrupted.^4^

The symptoms of MS vary widely from person to person. While some individuals experience only mild symptoms, others may suffer from severe impairments, including difficulty with vision, walking, and even speaking. Diagnosis typically involves when the symptoms are appeared by evaluating clinical symptoms and signs, along with imaging and laboratory tests.^4^

On the other hand, the nature of colorimetric biosensors is very suitable for them to be converted into rapid point-of-care (PoC) tests. Just by following the color change, these systems can give information about a disease present or absent in your system. Following the COVID-19 pandemic that was caused by the SARS-CoV-2 virus, the need for rapid, user-friendly economic and practical tests is fully understood. Since biosensors can be the core of rapid tests, the importance of these smart systems has increased recently.

Usually, the rapid test format-based biosensors depend on antigen–antibody, DNA, or aptamer interactions. However, there are other biosensors composed of glycan-based recognition reactions. Our group worked on these types of biosensors where the main reaction is to mimic the infection mechanism of the virus.^5^ In this way, we manage to produce both electrochemical and spectroscopic biosensors that can be used for infectious disease diagnosis.^5,6^ In the present study, we manage to construct a colorimetric and/or spectroscopic EBV biosensor that we believe will be more suitable to be converted into a rapid, PoC test for the future. Considering the link between EBV and MS, with this biosensor, we aimed to detect the presence of EBV in saliva even before the MS symptoms emerge. The reason we applied the developed EBV biosensor to saliva is that EBV infection typically begins in the saliva and primarily targets naïve B cells in the oropharynx and tonsils of the host.^7^ During this process, EBV transmission is believed to occur not only to memory B cells but also to epithelial cells. Importantly, EBV establishes lifelong persistence within the host once infection occurs.^7,8^

Several challenges currently limit the widespread application of the existing methods for EBV diagnosis. For instance, ELISA involves multiple detection steps and extended incubation times and carries the risk of false positives due to antigen–antibody cross-reactivity. Although PCR offers high sensitivity, it is not practical for routine use because it requires numerous analytical steps and is highly susceptible to cross-contamination. Similarly, antibody detection in saliva is hindered by the slow result turnaround, making it less suitable for situations where rapid diagnostics are essential. Considering these limitations, there remains a clear need for the development of EBV diagnostic tools that are practical, rapid, accurate, sensitive, and selective—especially for early screening purposes.^9^

Like many other enveloped viruses, EBV initiates infection by binding its viral glycoproteins to specific receptors on the host cell surface. However, unlike most similar viruses, EBV utilizes a variety of glycoproteins to interact with multiple host cell receptors. For instance, the envelope glycoprotein gp350/220 binds to CD21 or CD35 on B cells, facilitating viral attachment and entry.^7,8,10^ The mechanism of this attachment involves the binding of gp350/220 to CD21 through its N-terminal region, spanning amino acids 1–470, which includes a glycan-free surface patch.

The predicted interaction site on gp350 is characterized by a highly negative electrostatic potential, whereas the SCR1 and SCR2 domains of CD21 display a positively charged surface. This charge complementarity strongly suggests that electrostatic forces contribute significantly to the binding interaction. Furthermore, the morphological features of the two surfaces appear to be structurally complementary. Nevertheless, direct structural data for the gp350–CD21 complex remain unavailable, and the precise molecular determinants of their interaction have not yet been fully elucidated.^7,8,10^

Meanwhile, AuNPs have attracted significant attention due to their high surface-area-to-volume ratio, excellent electrical conductivity, strong plasmonic properties, and ease of surface functionalization, enabling their integration into both advanced sensing and electronic platforms.^11^ Recent studies demonstrate that tailoring the AuNP surface chemistry can markedly enhance their functional performance. For example, Wang et al. utilized charged-ligand-functionalized AuNPs to generate tunable counterion gradients, thereby modulating electron transport and enabling the construction of diodes, transistors, memristors, and chemiresistive sensors with high flexibility, low power consumption, and operational stability in humid environments.^12^ In plasmonic biosensing, AuNPs have been extensively employed to improve the sensitivity and selectivity through their strong light–matter interactions and biocompatibility. Çimen et al. developed cortisol-imprinted SPR sensors in which AuNPs acted as signal amplification tags, achieving rapid, real-time detection with an ultralow detection limit of 0.0087 ppb.^13^ Similarly, Eriş et al. fabricated lysozyme-imprinted SPR chips decorated with AuNPs, integrating them into molecularly imprinted polymer matrices to enhance analyte–sensor interactions, yielding a detection limit of 0.008 μg/mL and recovery rates of approximately 99% in complex biological media.^14^ Beyond SPR-based approaches, Zhang et al. introduced weakly ionized, ascorbic acid-coated AuNPs in lateral flow immunoassays, improving antibody adsorption and activity while avoiding electrostatic aggregation, which led to an ∼100-fold increase in sensitivity and enabled naked-eye detection of trace biomarkers, including the differentiation of viral variants.^15^ Collectively, these findings underline that the rational engineering of AuNP surface chemistry—through ligand functionalization, molecular imprinting, or controlled ionization—offers powerful strategies to advance the sensitivity, selectivity, and versatility of next-generation diagnostic and nanoelectronics systems.

In this work, by mimicking the infection mechanism of EBV, AuNPs were functionalized with CD21. The main aim of this study is to fabricate an effective EBV diagnostic tool suitable for early and asymptomatic screening of the virus and hence MS. To the best of our knowledge, this is the first spectroscopic biosensor study to utilize the infection mechanism as the basis for EBV detection. After the formation of the bioactive layer, the reaction between CD21 and EBV protein gp350 occurs. This interaction could be monitored with the naked eye, as well as with a suitable spectrophotometer. After the operating parameters of EBV biosensors were optimized, the analytical characteristics were investigated. Finally, the performance of the developed spectrophotometric biosensor was tested by using saliva samples collected from healthy individuals.

## Experimental Section

### Materials

AuNPs with a diameter of 10 nm were obtained from MERCK. Recombinant human CD21 and recombinant EBV gp350 proteins were purchased from Sino Biological Inc. All reagents and chemicals employed in this study were analytical grade and used without further purification. All solutions were prepared with ultrapure water obtained from an Elektromag M4 ultrapure water system.

### Instrumentation

All experimental measurements were performed using a T60UV model ultraviolet–visible (UV–vis) spectrophotometer from PG Instruments LTD., with 3.5 mL of 10 mm^2^ quartz cuvettes. The pH of all buffer solutions, which were subsequently employed in the preparation of biological materials, was adjusted using a Thermo Detection Corporation pH meter. Solution preparation and mixing were performed using a Bandelin Sonorex ultrasonic bath and a Velp Scientifica vortex mixer, respectively.

Atomic force microscopy (AFM) analyses were performed in tapping mode at resonant frequencies ranging from 15 kHz to 29 kHz. All AFM results were obtained in air under ambient conditions. Meanwhile, with scanning electron microscopy (SEM), results were obtained after the specimens were sputter-coated with a 9 nm gold/palladium (Au/Pd) layer using a Leica EM ACE600 sample coater. SEM imaging was conducted at a chamber pressure of 1.00–3 Pa, with a resolution of 1 nm at an accelerating voltage of 1 kV. Infrared spectra were obtained in attenuated total reflectance mode using a Nicolet iS50 Fourier Transform Infrared (FTIR) spectrometer (Thermo Scientific, USA) over the wavenumber range of 4000–1 cm^–1^.

All incubation procedures were performed using a BIOSAN ES-20 environmental shaker incubator.

### Fabrication and Spectroscopic Analysis of the EBV Immunosensor

For the preparation of the EBV spectroscopic biosensor, 2.1 mL of commercial AuNPs was initially mixed with 400 μL of CD21 receptor at a concentration of 20 ng/mL. The mixture was then incubated at 25 °C for 30 min. Subsequently, 100 μL of 1% bovine serum albumin (BSA) was added, which was followed by incubation at 25 °C for 15 min. Finally, 200 μL of EBV gp350 protein at a concentration of 50 ng/mL was introduced, and the mixture was incubated at 37 °C for 60 min. All experiments were conducted within the wavelength range of 300–700 nm, with continuous mixing during the incubation periods to ensure homogeneity. The working principle of the developed spectroscopic biosensor is based on absorbance changes resulting from the interaction of the reagents with AuNPs. AuNPs exhibit a characteristic absorption peak at around 520 nm. In this study, AuNPs were initially functionalized by CD21. Then, this functionalized mixture interacted with the gp350 protein. These processes resulted in a decrease in the absorbance at 520 nm. By correlating this decrease with the concentration increment, quantitative analysis of gp350, hence EBV, was made. All spectroscopic measurements were performed in triplicate to ensure accuracy and reliability (Scheme 1).

**Scheme 1:**
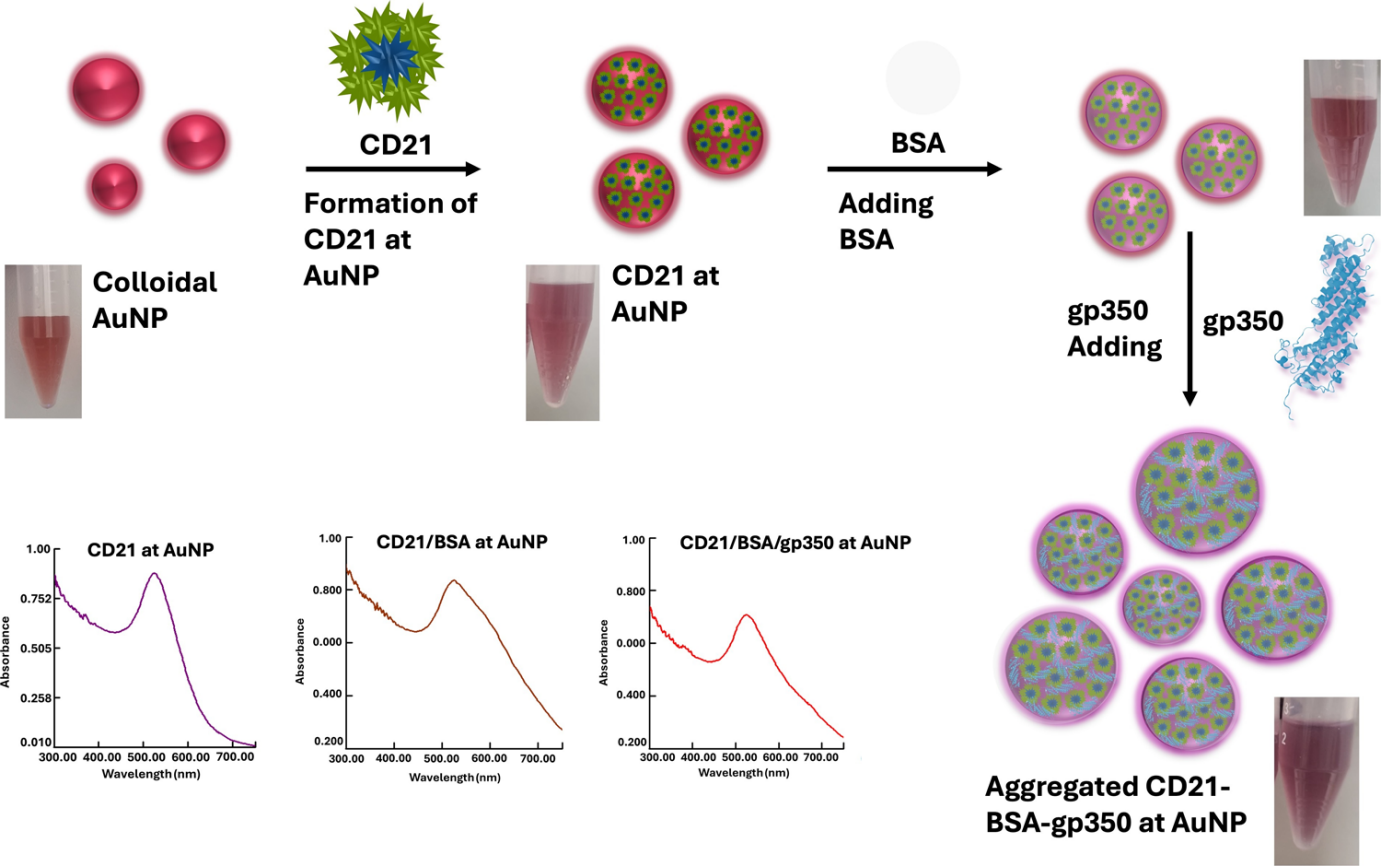
Schematic Representation of the Colorimetric/Spectroscopic EBV Immunosensor

### Interference Studies

For the interference studies, a gp350 concentration of 50 ng·mL^–1^ was employed, with potential interferents introduced at an equimolar ratio (1:1) relative to that of gp350. The A27L antigen from the Vaccinia virus and the KMP11 protein from Leishmania were identified as potential interfering agents for this study.

#### Real-Sample Application

Sample analysis was performed by spiking defined concentrations of gp350 into saliva samples diluted to 1:100. Saliva was collected from healthy individuals following oral hygiene procedures (tooth and mouth cleansing) and at least 2 h after their last meal. Prior to collection, each individual chewed pure, sugar-free gum for 5–10 min within the 2 h precollection window. gp350 was added to the saliva samples at concentrations of 5, 10, 25, and 50 ng·mL^–1^. Three separate saliva samples were collected, and each concentration was tested in triplicate. The resulting data were compiled and presented as a calibration curve.

All experimental procedures were reviewed and approved by the Ethics Committee of Mula Sıtkı Koçman University, Medical and Health Sciences (Protocol No. 240205 and Decision No. 160). The committee also waived the requirement of informed consent for this specific study. Nonetheless, we confirm that all methods were conducted in accordance with relevant ethical guidelines and regulations and that informed consent was obtained from all participants and/or their legal guardians, where applicable.

#### Storage Stability

The stability of the developed EBV biosensors was checked by following the absorbance of the seven solutions that were prepared by adding the CD21 receptor to the bare AuNP. For this purpose, biosensor performance was evaluated at defined time intervals following the addition of gp350 to the solution. Measurements were conducted daily for three consecutive days and subsequently on a weekly basis for a total duration of 1 month. Between measurements, all solutions were stored at 4 °C.

## Results and Discussion

### Characterization of Fabricated EBV Biosensor

In addition to spectrophotometric evaluations, the structural morphology of the spectroscopic EBV biosensor was investigated by using SEM (Figure 1A–C) and AFM (Figure 1D–F) images. SEM images reveal distinct morphological changes after each functionalization step. Initially, CD21@AuNPs (Figure 1A) exhibited a relatively uniform and compact surface, indicating stable conjugation of CD21 molecules on the nanoparticle surface.^16^ Upon the addition of BSA as a blocking agent (Figure 1B), the surface became more textured and layered, suggesting effective coverage of unoccupied sites by BSA and prevention of nonspecific adsorption.^17^ Following the specific interaction between the immobilized CD21 and viral gp350 protein (Figure 1C), a notable increase in surface roughness and visible aggregations was observed, indicating successful receptor–ligand binding and protein complex formation. These findings were further validated by AFM topographic imaging (Figure 1D–F). The CD21@AuNP surface (Figure 1D) showed relatively smooth topography, which became increasingly heterogeneous after BSA blocking (Figure 1E) and significantly rougher and elevated upon gp350 binding (Figure 1F), confirming successful biomolecular interactions at each stage.^18,19^ Collectively, the consistent surface transformations observed in both SEM and AFM images provide strong evidence for the effective layer-by-layer construction of the EBV biosensor and support the mechanism underlying colorimetric and spectroscopic signal generation based on specific interactions.

**Figure 1:**
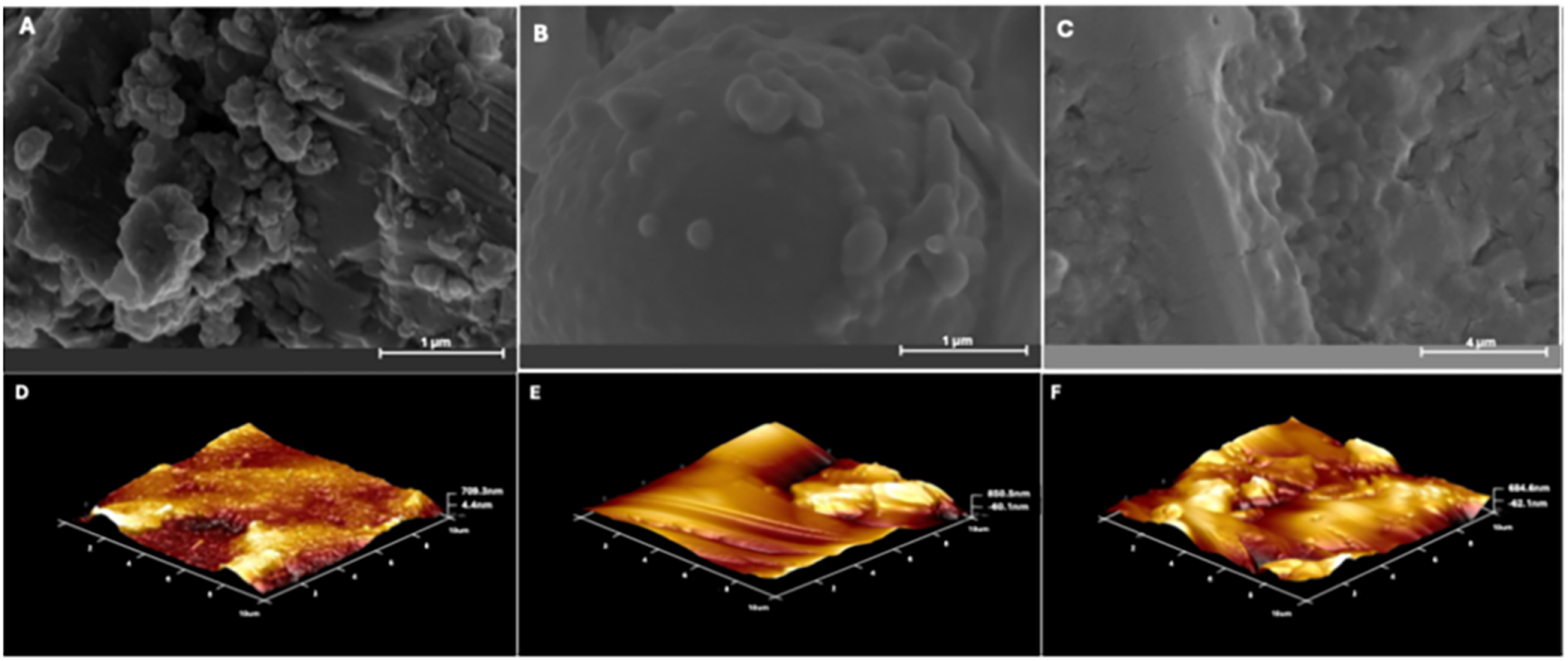
SEM (A–C) and AFM (D–F) images of (A, D) CD21@AuNP, (B,E) BSA@CD21@AuNP, and (C, F) gp350@BSA@CD21@AuNP.

Apart from structural characterization studies, FTIR spectra were provided in order to demonstrate the main interaction. The FTIR spectrum (Figure 2A) demonstrated bare AuNPs, CD21@AuNP, BSA@CD21@AuNP, and gp350@BSA@CD21@AuNP. The bands at ∼1680 and ∼3400 cm^–1^, corresponding to C=O and N–H stretching vibrations of amide bonds, while the signal at ∼500 nm belongs to the Au–O band.^20^ Additional peaks at 1636.98, 2100.00, and 3304.00 cm^–1^ confirm the presence of O–H bending, C–H stretching, and O–H stretching, respectively.^21^ The change in the broad band observed at approximately 3300 cm^–1^ when the proteinaceous substances were added onto AuNPs is attributed to the N–H stretching overlapped with the O–H stretching, indicating the presence of proteins and the formation of an extensive hydrogen-bonding network.^22,23^ While this band is weak in bare AuNPs, it becomes more pronounced in CD21@AuNPs and further increases in intensity for BSA@CD21@AuNPs and gp350@BSA@CD21@AuNPs. The amide I band (∼1650 cm^–1^; C=O stretching) and amide II band (∼1540 cm^–1^; N–H bending + C–N stretching) also become progressively more intense with each functionalization step, confirming protein adsorption.^24,25^ Notably, upon BSA conjugation, the amide I peak shifts from ∼1653 to ∼1644 cm^–1^ and exhibits broadening, which is consistent with literature-reported conformational rearrangements involving a decrease in the α-helix content and an increase in β-sheet/random coil structures.^26^ In bare AuNPs, the doublet at ∼1590–1560 and ∼1410–1380 cm^–1^ corresponds to the asymmetric and symmetric ν­(COO^–^) stretches of citrate, confirming the capping agent from the synthesis process.^27^ Finally, gp350 immobilization results in the greatest broadening of amide A and the highest intensity of amide I/II bands, validating that the multilayer bioconjugation process has been successfully completed, as corroborated by similar observations in AuNP–antibody systems.^25^

**Figure 2:**
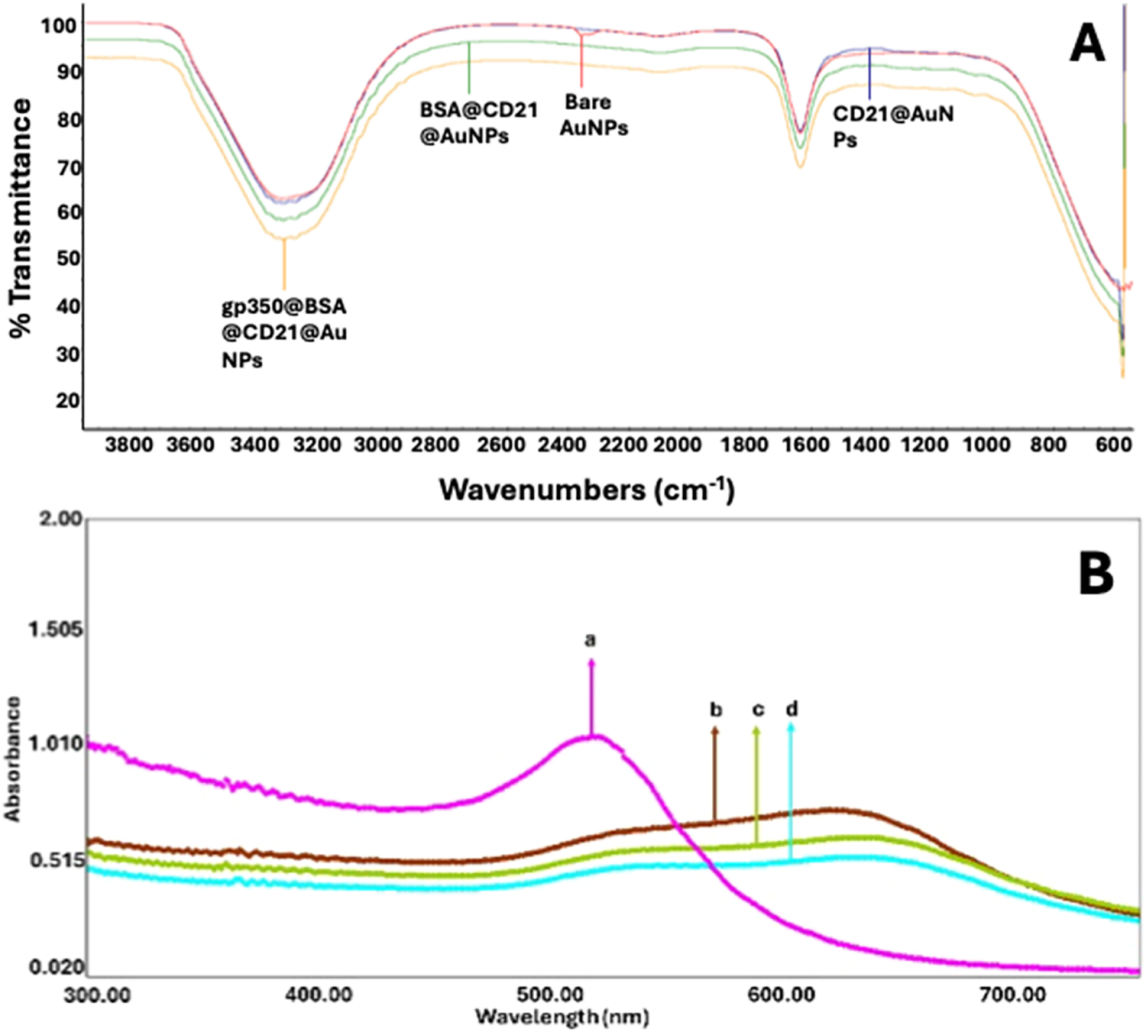
(A) FTIR and (B) UV spectra for the characterization of the developed colorimetric EBV biosensor. Step-by-step FTIR and UV spectra of the biosensor during gp350 detection: (a) 2.1 mL of AuNPs; (b) 2.1 mL of AuNPs + 400 μL of CD21 at 20 ng·mL^–1^; (c) 2.1 mL of AuNPs + 400 μL of CD21 at 20 ng·mL^–1^ + 100 μL of 1% BSA; and (d) 2.1 mL of AuNPs + 400 μL of CD21 at 20 ng·mL^–1^ + 100 μL of 1% BSA + 50 ng·mL^–1^ of gp350.

Moreover, the spectrophotometric characterization of the developed EBV biosensor had also been done (Figure 2B). From the figure, it is obvious that plain AuNPs have the biggest absorbance value in terms of the absorbance spectrum since no layer has been formed onto them. Upon immobilization of CD21, a decrease and a shift in the absorbance value were seen as shown in Figure 2B­(b). Each sequential bioconjugation step led to a progressive decrease and shift in absorbance values observed in the colorimetric spectrum, attributed to the gradual coverage of the AuNP-based sensing surface with biomolecular layers. This decrease reflects a reduction in light absorption, likely due to conformational changes and increasing surface shielding by the immobilized proteins within the colloidal solution. Upon the immobilization of BSA onto the CD21-functionalized AuNPs, a further decline in absorbance was recorded, indicating efficient surface blocking and suppression of nonspecific binding. Finally, the introduction of gp350—mimicking the EBV infection mechanism—produced an additional, expected drop in the absorbance signal. This response confirms the occurrence of a specific molecular interaction between gp350 and CD21, thereby validating the functionality and sensitivity of the biosensor platform.

As is well known, AuNPs exhibit higher reactivity than many other metals due to their elevated surface energy, making them highly interactive with biomolecules such as proteins, lipids, and nucleic acids. These interactions generally occur through noncovalent forces—such as van der Waals forces, hydrophobic interactions, electrostatic attractions, and hydrogen bonding—without the need for covalent bond formation or cleavage.^17,28,29^ Such interactions significantly influence the biological reactivity of nanoparticles, including AuNPs.^17^ A common result of these interactions is the formation of a protein corona—a dynamic, membrane-like layer formed by the adsorption of proteins and other macromolecules onto the nanoparticle surface over time.^17,30^ In this study, it is hypothesized that the high concentration of the CD21 receptor added to the system was adsorbed onto the AuNP surface during incubation, leading to protein corona formation.^30^

Meanwhile, the working principle of the developed EBV biosensor was based on the localized surface plasmon resonance (LSPR) properties of AuNPs.^31^ The LSPR spectrum of AuNPs is highly sensitive to changes in particle size and aggregation state.^31–33^ Typically, AuNPs exhibit a characteristic absorption peak around 520 nm.^34^ Upon functionalization of the AuNP surface with CD21, the gp350 protein specifically interacted with CD21. This interaction triggered nanoparticle aggregation, which increased particle size and caused a red shift in the LSPR band. Visually, this appeared as a color change from pink-red to purple. Additionally, the decrease in absorbance at 520 nm correlated with a more transparent solution, indicating successful binding and aggregation.^34,35^

### Working Condition Optimization Studies

After the CD21 amount was selected based on the literature,^5^ in order to obtain better accuracy and hence enhancing the developed biosensor performance, CD21 and gp350 incubation temperature and incubation time optimizations have been performed.

### Incubation Temperature Optimization Studies

It is well established that temperature is a critical physicochemical parameter that markedly influences protein structure and function. Proteins may exhibit altered activity profiles under varying thermal conditions.^36^ In accordance with this principle, a series of incubation temperatures (4, 25, 37, and 45 °C) was systematically evaluated to optimize the interaction between CD21 and gp350 by utilizing a colorimetric analytical approach. Among the tested conditions, the incubation at 37 °C yielded the most pronounced absorbance change, indicating maximal interaction efficiency (Figure 3). Protein conformational flexibility in response to thermal variations is particularly significant in domains responsible for molecular recognition and catalytic activity. It has been previously reported that thermally induced structural perturbations within these functional domains may impair protein–ligand binding capacity and overall bioactivity.^36^ This phenomenon is likely responsible for the reduced colorimetric signals observed at suboptimal temperatures.

**Figure 3:**
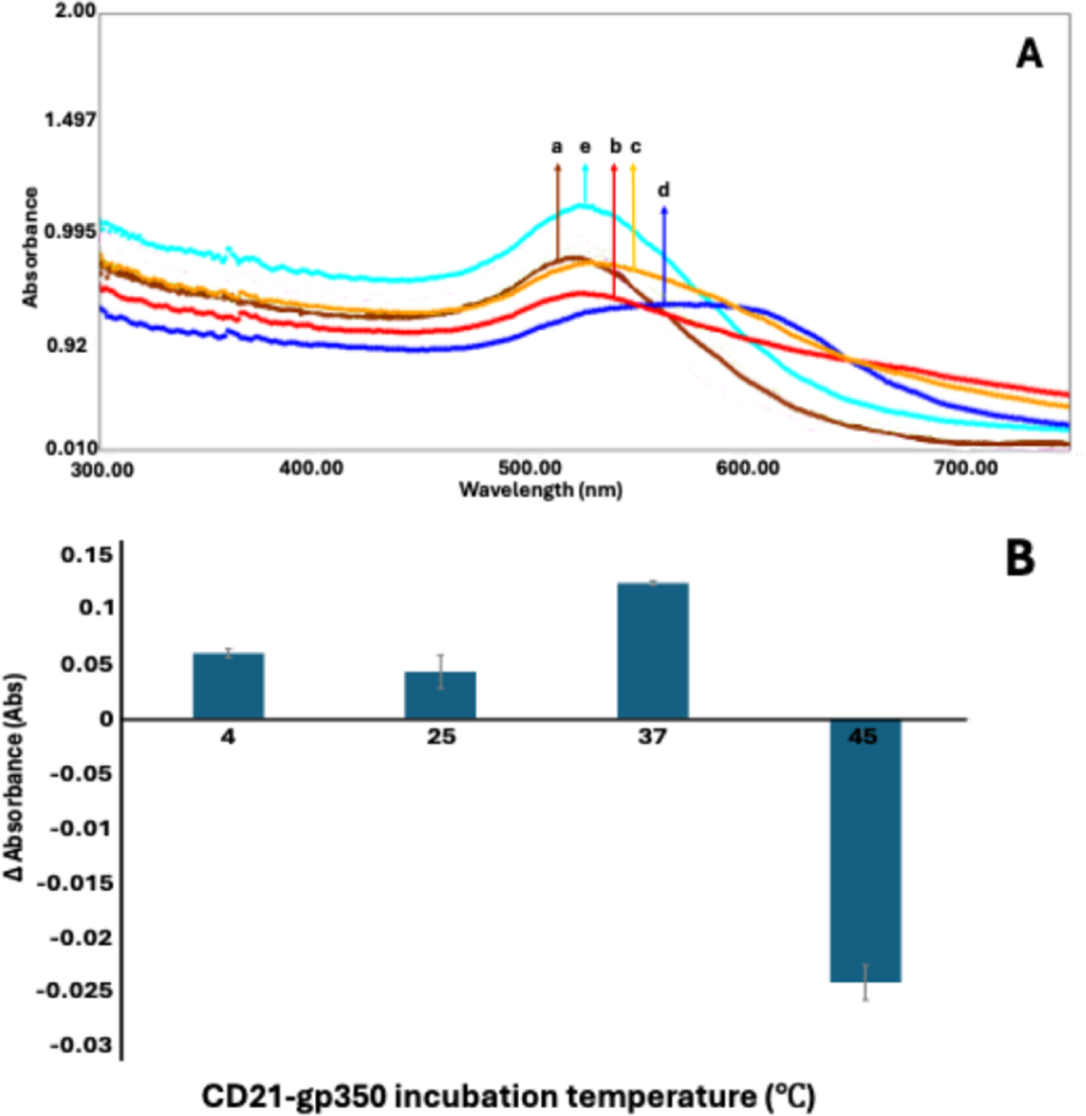
(A) UV spectra and (B) bar chart that belong to the optimization of the CD21–gp350 interaction incubation temperature. (a) BSA@CD21@AuNP, (b) BSA@CD21@AuNP + 50 ng·mL^–1^ gp350 for 4 °C, (c) 25 °C, (d) 37 °C, and (e) 45 °C incubation temperature.

### Optimization of CD21–gp350 Incubation Time

The optimization of the incubation duration plays a pivotal role in enhancing the sensitivity and overall analytical performance of colorimetric or spectroscopic biosensors. To determine the most suitable incubation time for the developed EBV biosensor, various durations (15, 30, 60, and 90 min) were systematically tested. As illustrated in Figure 4, the absorbance signal reached its peak at 60 min, indicating the most efficient biomolecular interaction within this time frame. Among the tested intervals, 60 min provided a balance between analytical efficiency and experimental practicality. Therefore, this incubation time was designated as the optimal condition and was employed in subsequent experimental procedures.

**Figure 4:**
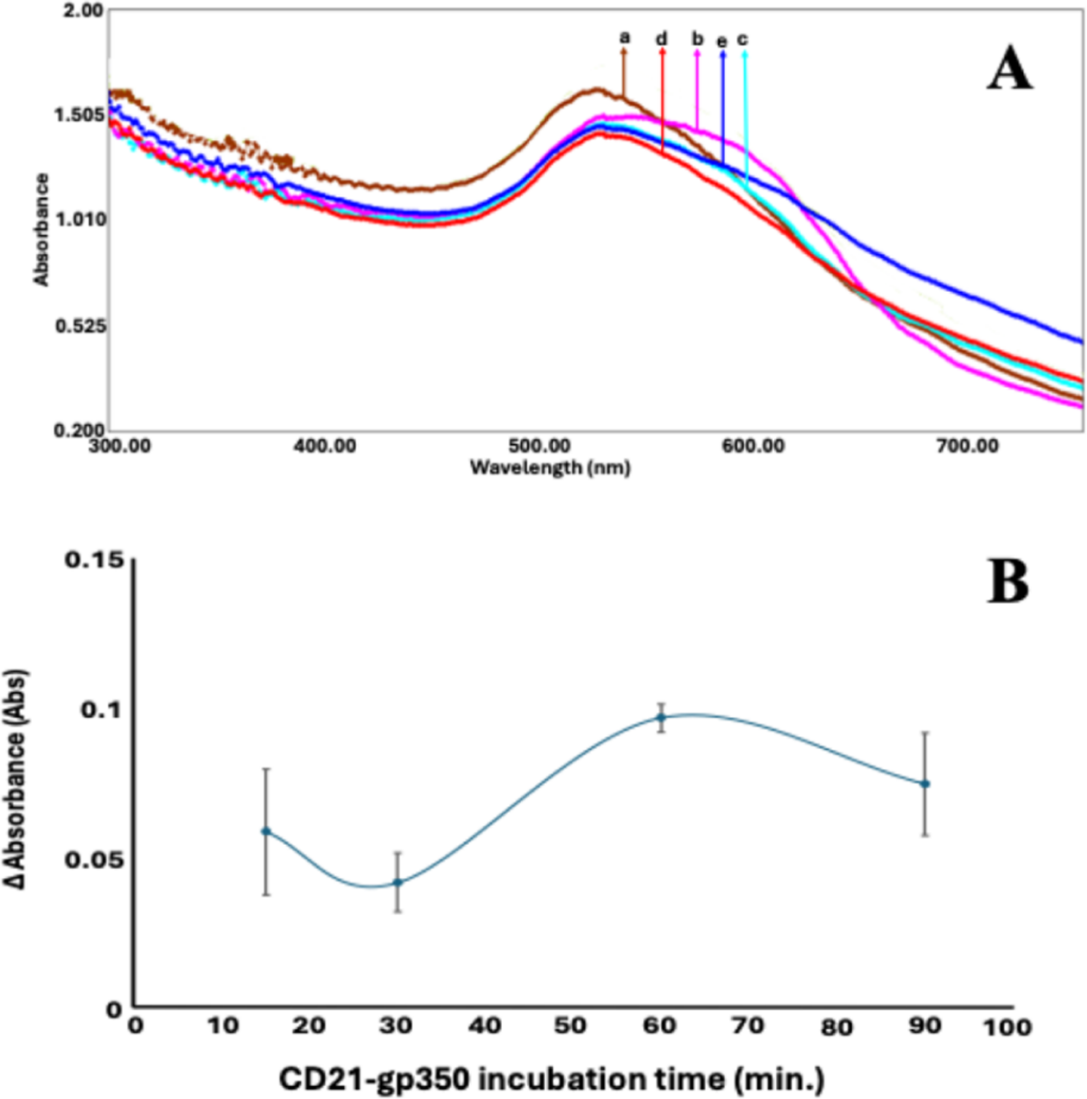
(A) UV spectra and (B) Excel plot that belong to the optimization of the CD21–gp350 interaction incubation time. (a) BSA@CD21@AuNP, (b) BSA@CD21@AuNP + 50 ng·mL^–1^ gp350 for 15 min, (c) 30 min, (d) 60 min, and (e) 90 min incubation time.

### Analytical Characteristics

The analytical characteristics of the developed EBV immunosensor were examined under the optimal working conditions. As presented in Figure 5B, the linear range was obtained between 10 and 100 ng mL^–1^ concentration range with the equation of *y* = 0.0021*x* + 0.0264 (*R* ^2^ = 0.9971). Limit of detection (LOD) and limit of quantification (LOQ) values, which were two indicators of the sensitivity of the systems, were also calculated. For the calculation of LOD value, a 3.3 s/m^37^ formulation was used, whereas 0.846 represents the standard deviation of the blank, and m: 0.9972, the slope of the calibration curve, while for LOQ calculation, 10 s/m equation was utilized.^37^ As a result, the LOD and LOQ values were found as 0.0279 and 0.0848 ng/mL^–1^, respectively. Apart from these, a relative standard deviation value that associates with the repeatability was also calculated for 50 ng/mL^–1^ gp350 and found as 0.848% (*n* = 3).

**Figure 5:**
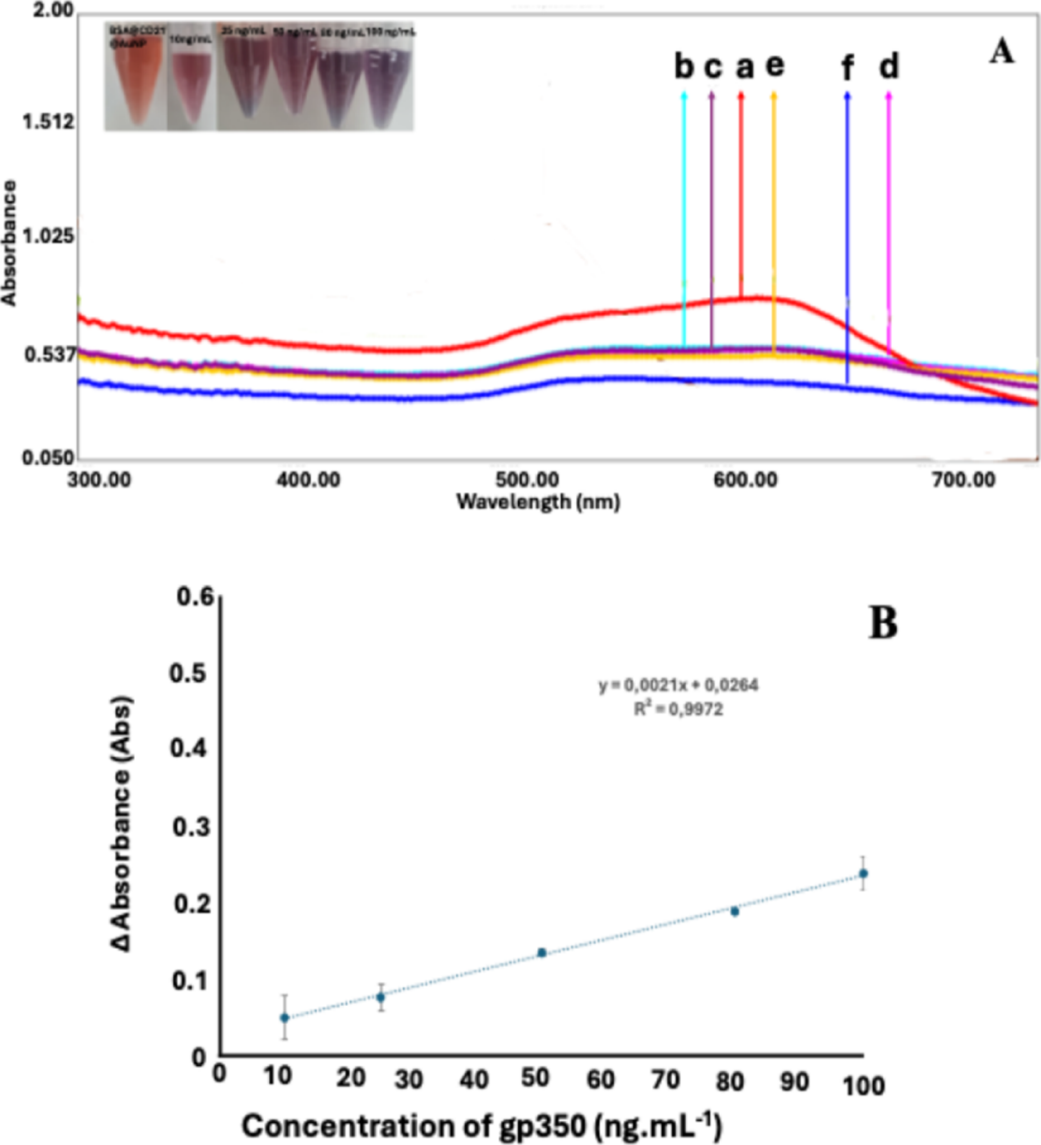
Analytical characteristics of the developed colorimetric EBV biosensors. (A) UV spectra of (a) BSA@CD21@AuNP, (b) 10 ng·mL^–1^, (c) 25 ng·mL^–1^, (d) 50 ng·mL^–1^, (e) 80 ng·mL^–1^, and (f) 100 ng·mL^–1^ gp350 additions. (B) Calibration plot of increasing concentrations of gp350 from 10 ng·mL^–1^ to 100 ng·mL^–1^.

The storage stability of this EBV immunosensor was also searched. After 1, 2, 15 (2 weeks), and 30 (one month) days, the recovery values were found as 97.10, 99.28, 97.82, and 91.30%, respectively (data not shown).

### Interference Study

The effects of selected interferents were searched for 50 ng·mL^–1^ gp350 under optimal experimental conditions, where potential interferents were introduced at an equimolar ratio (1:1) relative to gp350. The A27L antigen of the Vaccinia virus and the KMP11 protein found in Leishmania were identified as potential interfering agents. Absorbance signals of these 1:1 cocktail mixtures yielded a recovery value percentage of 108.2% (*n* = 3). These results confirm the accuracy and applicability of the EBV biosensor, even in the presence of selected interferents (Figure 6).

**Figure 6:**
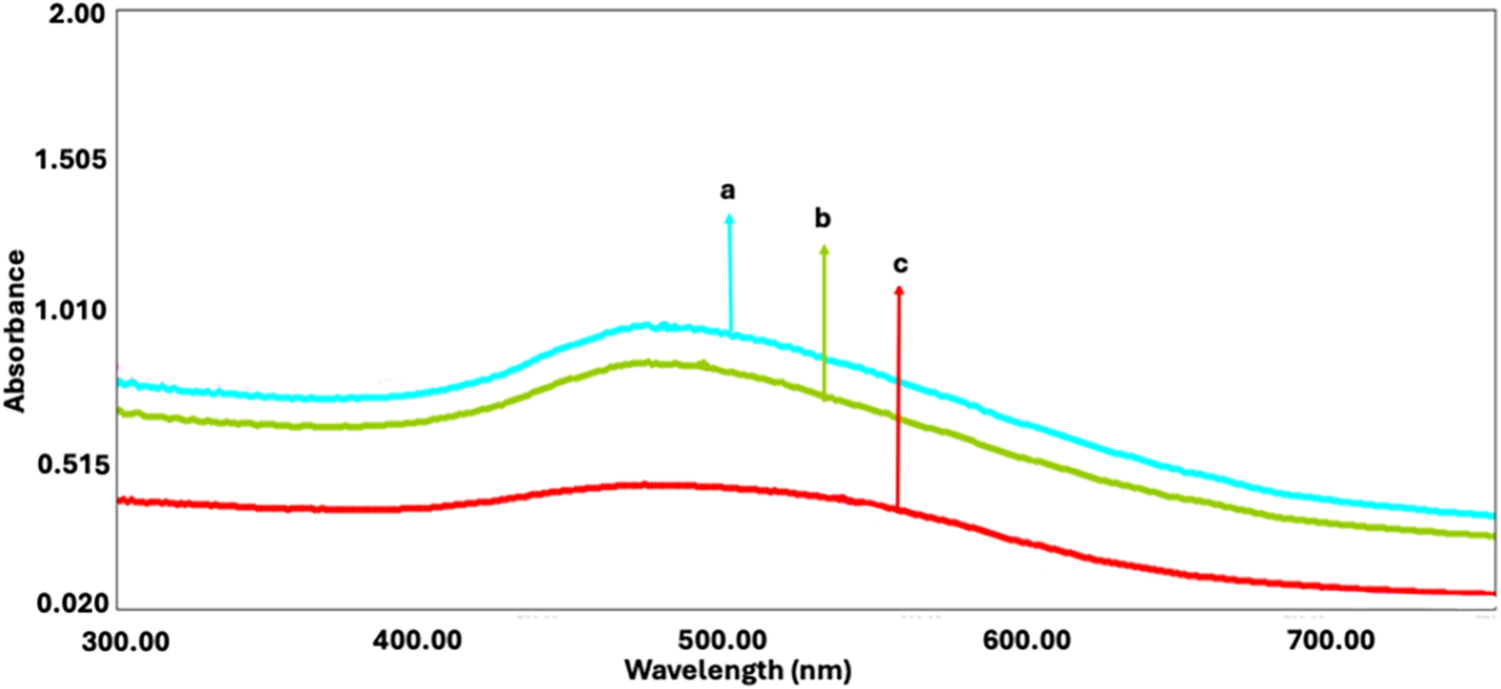
UV spectra demonstrating the absorbance difference of a 1:1 ratio of (a) BSA@CD21@AuNP, (b) gp350 @BSA@CD21@AuNP, and (c) A27L + KMP11 + gp350 @BSA@CD21@AuNP.

### Sample Application

For a sample application, properly diluted saliva samples from healthy individuals were used. For conducting the study, a series of gp350 solutions, such as 10, 25, 50, and 100 ng mL^–1^, were prepared in a 1:100 diluted saliva. The effect of the saliva medium on the developed biosensor response was observed via UV–vis spectra (Figure 7). As can be seen from the figure, a correlation was obtained between increased gp350 concentration and absorbance values. Also, when compared to the buffer-included calibration studies’ absorbance values, the following percentages such as 110.2, 90.66, 97.61, and 103.37% were obtained for 10, 25, 50, and 100 ng mL^–1^, respectively.

**Figure 7:**
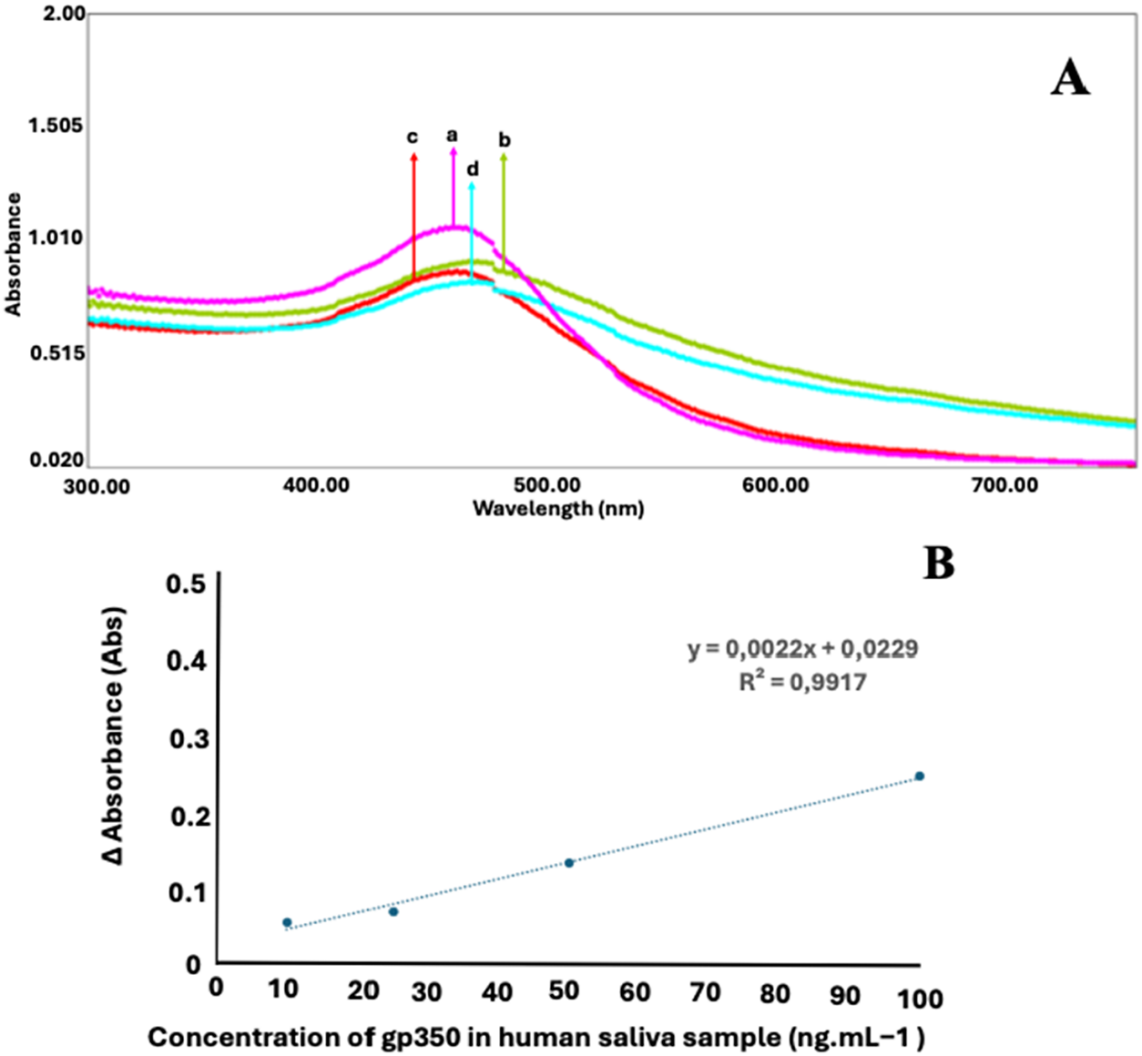
UV spectra (A) and Excel plot (B) regarding (a) 10 ng mL^–1^, (b) 25 ng mL^–1^, (c) 50 ng mL^–1^, and (d) 100 ng mL^–1^ gp350 included 1:100 diluted saliva.

## Conclusions

The goal of this study is to develop an early screening tool that enables the asymptomatic detection of EBV infections, hence the early detection of MS. In the population, EBV often presents with either no symptoms or symptoms that are indistinguishable from other mild, self-limiting illnesses. Considering analytical characteristics like linear range, LOD, LOQ, and RSD values together with recovery values that were obtained with real human saliva, it can be concluded that this biosensor system may therefore offer a promising method for early, noninvasive diagnosis. On the other hand, we believe that this biosensor system has the potential to be converted into a rapid test for the future. Also, considering its spectroscopic, hence colorimetric nature, it will be easy to adapt the developed EBV immunosensors into lateral flow assays or PoC colorimetric assays for the future.
